# Azurite in medieval illuminated manuscripts: a reflection-FTIR study concerning the characterization of binding media

**DOI:** 10.1186/s40494-019-0262-1

**Published:** 2019-04-03

**Authors:** Wilfried Vetter, Irene Latini, Manfred Schreiner

**Affiliations:** Institute of Science and Technology in Art, Academy of Fine Arts Vienna, Schillerplatz 3, 1010 Vienna, Austria

**Keywords:** Illuminated manuscripts, Non-invasive, Reflection-FTIR, Azurite, Binding medium, Glair, Egg white, Arabic gum

## Abstract

In illuminated manuscripts, a reliable identification of oxyanion pigments such as azurite by rFTIR is simple, as several combination and overtone bands are strongly enhanced compared to transmission mode. However, the characterization of the used binding media is rather difficult, as the analysis of four medieval manuscripts from the late thirteenth to the fifteenth century (e.g. Cod. slav. 8 in the collection of the Austrian National Library), as well as the earliest known map of Vienna (Albertinischer Plan from 1421, Wien Museum) showed. According to the literature, mainly glair (egg white) and plant gums were applied as binding media for azurite. Moreover, both were used in many cases also as “varnishes” in order to improve optical and mechanical properties of the paint layer. In order to assess the possibilities and to distinguish between proteinaceous and carbohydrate binders, mock-ups with azurite were prepared on parchment support with various quantities of binders. Additionally, some of the specimen were varnished using the binders mentioned above. Furthermore, mock-ups on aluminium foil were prepared to evaluate the influence of the support on the reflection spectra. The results showed that the binding medium content in the mock-ups usually was too low for a reliable determination by rFTIR (except the ones with the highest contents), whereas it was possible to characterize the varnish materials. Only an insignificant influence of the support on the spectra from the mock-ups was observed. However, the spectra obtained from three manuscripts suggested a certain influence of the parchment support, which indicates thinner paint layers.

## Introduction/background

Azurite (Cu_3_(CO_3_)_2_(OH)_2_), is composed of basic copper carbonate. The mineral azurite is found in many parts of the world in the upper oxidized portions of copper ore deposits. Azurite mineral is usually found in association with malachite (Cu_2_CO_3_(OH)_2_), the green basic carbonate of copper, and often with other copper-rich minerals (e.g. cuprite, tenorite, and chrysocolla) [1]. Depending of the region and the time period, the pigment was known under different names such as azzurro della magna, azzurro citramarina (Italy) [2, 3], Bergblau (Germany), bleu de montagne or bleu d’Allemagne (France) [4], blew bice (England) [2], lapis armenius (Pliny) or Berglasur (Agricola) [4], as well as several other names referring to the origin (German azure, Spanish blue, Hungarian blue, Ragusa blue, Lombard blue) [5]. The natural pigment, which was widely used in manuscripts, as well as panel and wall paintings, was made by grinding it to a powder and washing with plain water or solutions containing soap, gum and lye [5]. Furthermore, solutions containing honey, fish glue or gum were used [1]. The optical quality of the resulting pigment depends on the content of impurities and even more on the particle size. Deep blue qualities, which were favored in medieval times, are relatively coarse and the pigment turns pale if it is ground too fine. A larger particle size leads to longer absorption paths of the incident light before reflection and thus a darker appearance, but on the other hand the applicability is becoming worse and it might be too gritty to be used as a pigment [5]. The most important binding media for manuscript illuminations were clarified egg white or glair (clare, albumen, glarea, albuginea ovi) and gums, e.g. Arabic gum (gumma). Furthermore, fish glue (ichtyocollon), parchment size (cola pergamena), and casein glue (glutine casei) were used as well. In most cases only a single binder was applied, but depending on the technique or pigment, mixtures were also prepared in different proportions [6, 7] and it is reported that glair and occasionally Arabic gum [6] were used for varnishing in order to improve the optical and mechanical properties of the paint layers.

The availability of mobile analytical instruments, which are applicable in a non-invasive way, as well as an enhanced collaboration between natural sciences, humanities, and computer sciences has led to an increased analytical interest in illuminated manuscripts in the last years [8–14]. These investigations aim to characterize the materials in the manuscripts and to elucidate the techniques which were applied for their production. Among the analytical techniques used for these purposes, Fourier transform infrared spectrometry in the reflection mode (rFTIR) has proved to be suitable for the characterization of several inorganic and organic materials encountered in manuscripts, such as the parchment support, pigments, contaminants, or degradation products [9–16], although the evaluation of reflection data is often complicated by the contribution of both, specular and diffuse reflection [10]. Nevertheless, the literature shows that azurite can easily be identified by several combination bands in the rFTIR spectrum, which are strongly enhanced compared to spectra obtained in transmission mode, and that the binding medium usually cannot be characterized, except in one case [16].

We were also able to identify azurite in several medieval manuscripts on parchment during investigations carried out within the framework of CIMA (Centre of Image and Material Analysis in Cultural Heritage) [17] without receiving significant information concerning the binding medium. Although some of the spectra showed spectral features of proteins, it was not clear whether they derive from a proteinaceous binding medium or the parchment support. For this reason, we examined mockups with paint layers of azurite with either egg white or Arabic gum on parchment in order to assess the possibilities for a reliable characterization of proteinaceous and polysaccharide binding media using rFTIR. In addition, the resulting paint layers were partially varnished by egg white or Arabic gum to test the influence of this treatment and furthermore, mockups on aluminum foil were prepared in order to evaluate the influence of the support on the resulting rFTIR spectra. The present study should contribute to the knowledge about historical painting techniques and a better understanding of FTIR spectra obtained in reflection mode.

## Experimental

### Manuscripts

**Fig. 1:**
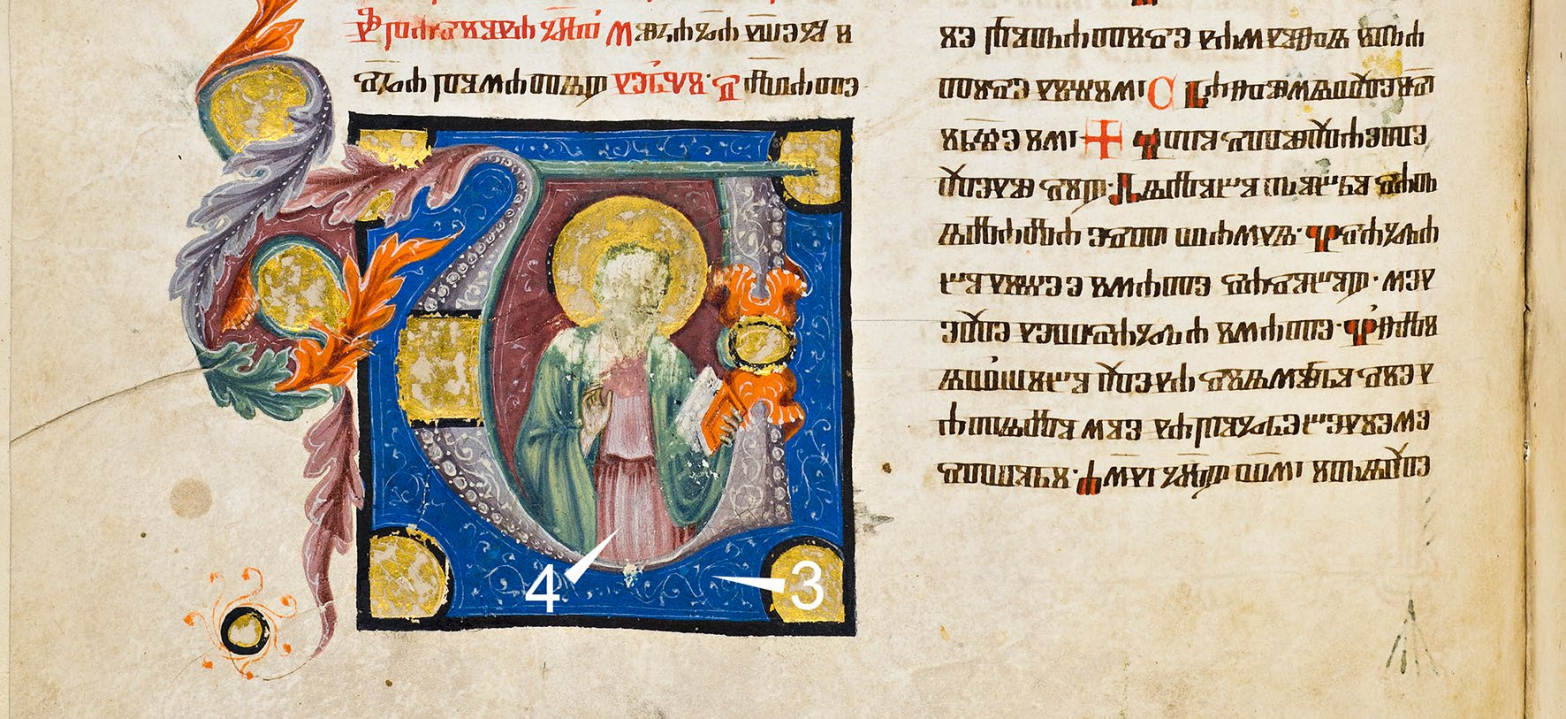
Detail from folio 74 verso in Codex slavicus 8 in the Austrian National Library, which contains various illuminations and decorations of outstanding quality

Four richly illuminated manuscripts with azurite findings (all on parchment), were analyzed in the libraries and museums where they are kept. Codex slavicus 3 (A.D. 1396) and Codex slavicus 8 (A.D. 1368) in the Austrian National Library (Fig. 1), De Scientia Venandi per Aves (Latin Moamin, late thirteenth century) in the Kunsthistorisches Museum Wien (Imperial Armoury), and Liederhandschrift B (A.D. 1432) by Oswald von Wolkenstein in the University and State Library Tyrol. Furthermore, azurite was detected in the earliest known map of Vienna and Bratislava—Albertinischer Plan (A.D. 1421), kept in the Wien Museum Karlsplatz. The map was drawn and painted on paper, which allowed a comparison of different supports.

### Mockups

Azurite with a particle size of approximately 14 µm (Order number: 1673112) was purchased from Kremer Pigmente (Aichstetten, Germany); Arabic gum solution (Sennelier) with 20% dry mass content (Order number 8-33577) was obtained from Gerstaecker (Germany). The Arabic gum solution contains less than one per cent of a mixture of the preserving agents 5-chloro-2-methyl-1,2-thiazol-3(2H)-one and 2-methyl-1,2-thiazol-3(2H)-one, which did not lead to visible spectral features in transmission FTIR spectra of dried Arabic gum. Glair was produced from the egg white of fresh chicken egg, which was whipped to a stiff froth by use of a handheld electric mixer with stainless steel beaters. The froth was allowed to stand for 5 h and the watery liquid on the bottom of the vessel was collected for the experiments, which had a dry mass content of 12%. Calf parchment was provided by the National Research & Development Institute for Textiles and Leather, Bucharest (INCDTP). The paint layers were applied to the flesh side of the parchment, where only negligible contents of calcium carbonate were detected by rFTIR (in contrast to the hair side).

**Fig. 2:**
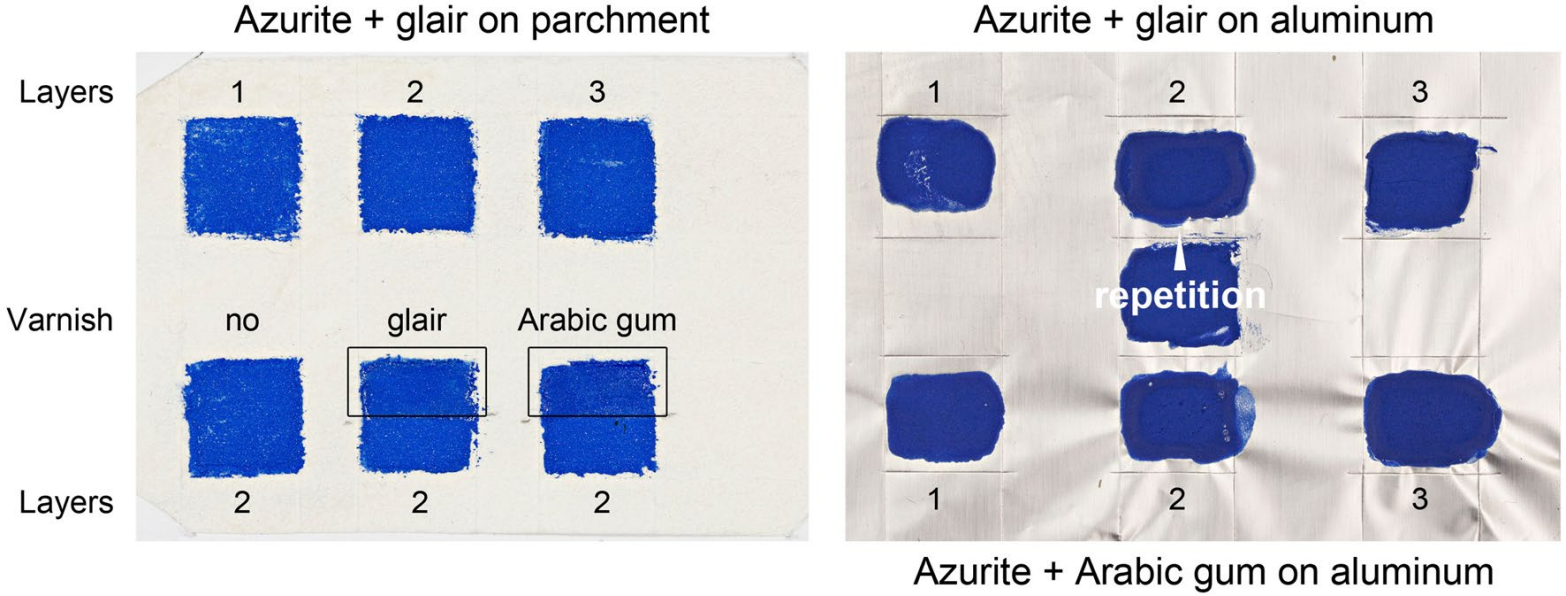
Azurite with glair as binding medium (one, two and three layers) on parchment and aluminum foil. Color patches with two paint layers were partially varnished

With regard to the practical applicability of the pigment/binder mixtures preliminary tests revealed that best results were obtained by mixing 0.5 g azurite pigment with approximately 300–400 µl binder solutions. It was not possible to obtain satisfactory results with either higher amounts of the aqueous binder solutions (quick sedimentation of the pigment particles), or lower amounts (mixture is too dry for an adequate application). Hence, the mockups were prepared by mixing either 0.5 g azurite, 300 µl glair and 100 µl water, or 0.5 g azurite and 300 µl Arabic gum solution, and the resulting paints were applied to the flesh side of the parchment with a 5 mm flat brush (Synthetic, Gerstaecker, Order number 8-73432) to produce color patches (1 cm × 1 cm) with one, two and three paint layers. A similar procedure was used to prepare mockups with household aluminum foil (Alufix) as support (Fig. 2). Additionally, color patches with two layers were partially varnished by applying either glair or the Arabic gum solution with the 5 mm brush.

Although the mentioned ratios between the pigment and the binders provided paint layers with very good mechanical and optical properties, the influence of various binding medium contents was tested. For this purpose, the Arabic gum solution was allowed to dry and the residue was dissolved in water to produce solutions with 50% and 35%. 300 µl of each solution were mixed with 0.5 g azurite to prepare mockups in the same way as described above. Glair was concentrated to 20% dry mass content by evaporation of a corresponding quantity of the water, and 400 µl of the resulting solution were mixed with 0.5 g azurite for the mockups.

For comparison, powder samples without binder were analyzed on glass microscope slides. The weight of a cover slide (0.17 mm thickness) was used to flatten the surface of the powder without additional pressure. Furthermore, glair and Arabic gum without pigment were applied on the parchment in order to assess their spectral features.

## Instrumentation

rFTIR measurements were performed using a spectrometer Alpha operated with software Opus 7.5 (Bruker Optics, Ettlingen, Germany), equipped with the module for external reflection. The instrument has a beam diameter of approximately 5 mm (roughly circular) and the background was acquired using a gold mirror. Reflection spectra were collected in the range between 4000 and 375 cm^−1^ (manuscripts), as well as 5000 and 375 cm^−1^ (mockups) with a resolution of 4 cm^−1^ over 64–128 scans. The reflection spectra were either directly evaluated, or occasionally after Kramers–Kronig algorithm, followed by baseline correction.

## Results and discussion

### Characterization of the azurite powder

**Fig. 3:**
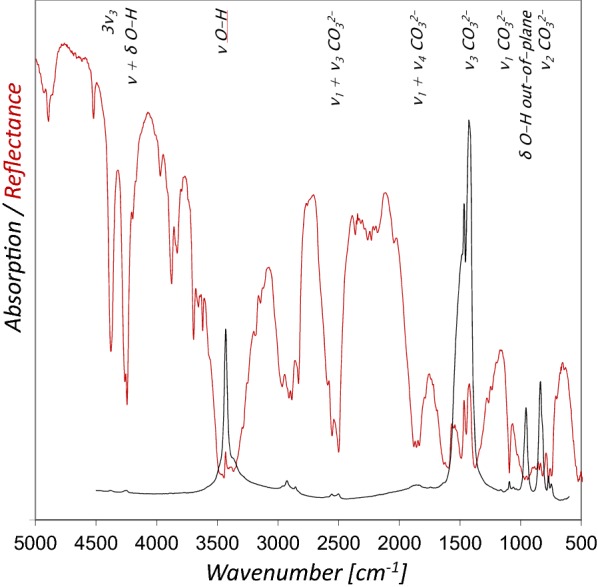
Comparison of the rFTIR spectrum of azurite 14 µm (red line) with the IRUG reference spectrum MP0001 (black line). The most important bands are indicated

**Fig. 4:**
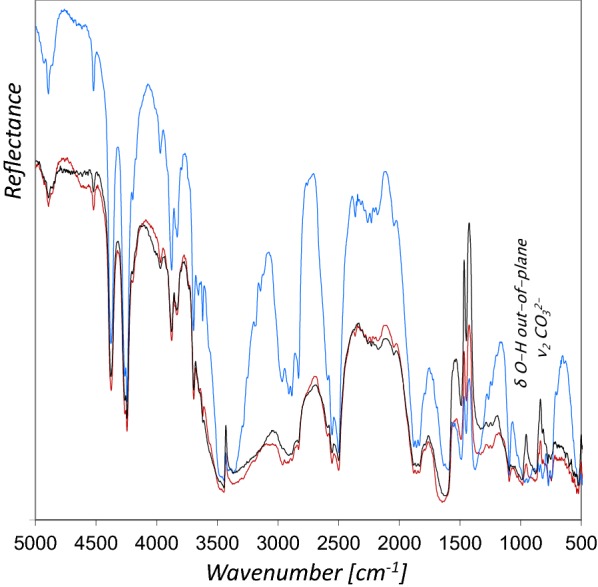
The rFTIR spectra of azurite 14 µm with glair (red line) or Arabic gum (black line) show additional spectral features compared to azurite without binder (blue line). The δ O–H out of plane bending and the ν_3_ CO_3_^2−^ bands are much stronger if a binder is added

The characteristic spectral features in the rFTIR spectrum of powdered azurite and their assignment according to the literature [10, 13, 18] are presented in Fig. 3. A comparison to the IRUG [19] reference database spectrum obtained in transmission mode yields that most of the bands show maxima at similar wavenumbers, although the O–H stretching (ν O–H, 3432 cm^−1^) and CO_3_^2−^ stretching (ν_3_ CO_3_^2−^, 1466 and 1423 cm^−1^) bands are inverted. The combination and overtone bands are in general strongly enhanced (3ν_3_ at 4376 cm^−1^, ν + δ O–H 4244 cm^−1^, 2ν_3_ + ν_1_ CO_3_^2−^ at 3970 cm^−1^, 2ν_3_ + δ O–H 3877 and 3832 cm^−1^, ν_1_ + ν_3_ CO_3_^2−^ at 2590, 2554 and 2499 cm^−1^) and hence enable the detection of even low quantities of azurite therefore [10]. Interestingly, the δ O–H out of plane bending and the ν_3_ CO_3_^2−^ bands are almost not to be seen in the rFTIR spectrum of the powder in Fig. 3, whereas both are clearly visible in the rFTIR spectra of the mixtures with either glair or Arabic gum as binding media (Fig. 4).

In addition to the bands of azurite, also the O–H stretching bands of kaolinite could be detected at 3696, 3655 and 3620 cm^−1^, whereas the Si–O stretching bands in the range of 1000–1100 cm^−1^, which potentially could interfere with the C–O stretching vibrations of the Arabic gum binder, were almost missing in the spectra obtained [20, 21]. However, a slight influence on the results from the mockups cannot be completely excluded. As described in the literature [22], natural azurite frequently contains impurities, particularly malachite and silicates, although kaolinite is not mentioned. The microscopic evaluation of the purchased azurite showed contents of white, greenish and red particles, which were not further analyzed.

### Mockup samples

Compared to the rFTIR spectrum of azurite powder, the addition of both, glair and Arabic gum resulted in additional spectral features in the range of 3700–3100 cm^−1^ (N–H and O–H stretching), as well as 1750–1600 cm^−1^ (Fig. 4). In case of Arabic gum as binder, no additional bands can be determined in the ν C–O range of 1160–900 cm^−1^.

The differences between the rFTIR spectra with either glair or Arabic gum are only marginal, and do not allow a reliable differentiation between both types of binders. Small differences are observed between 4800–4590 cm^−1^, 3400–3000 cm^−1^, and 1700–1600 cm^−1^. Carlesi and coworkers [23] used an instrument similar to ours (Alpha) to analyze model films of pure binding media on glass slides in the NIR range (7500–3900 cm^−1^) and were able to differentiate egg white and Arabic gum by application of multivariate analysis. This could be an interesting approach for further studies, although the situation is more complex when binders are mixed with pigments in mockups, or if various other components contribute to the spectra obtained from illuminations in historic manuscripts. Moreover, ageing processes of the materials might also complicate chemometric analyses. It has been further reported that a chemometric approach was successfully applied to discriminate transmission FTIR spectra of egg white and parchment glue that were used in reconstructions of medieval paints, exploiting the C–H stretching absorption regions [24]. However, the rFTIR spectrum of glair on parchment only shows one uncharacteristic, relatively broad C–H feature and that is also the case for Arabic gum. Furthermore, we frequently detected calcium soaps on the surface of paint layers in manuscripts, which also exhibit strong C–H bands. An example is presented in the following section, where the results obtained for Cod. slav. 8 are discussed. For these reasons, the application of a similar approach does not seem to be very promising in terms of the differentiation of glair and Arabic gum binders in illuminated manuscripts.

**Fig. 5:**
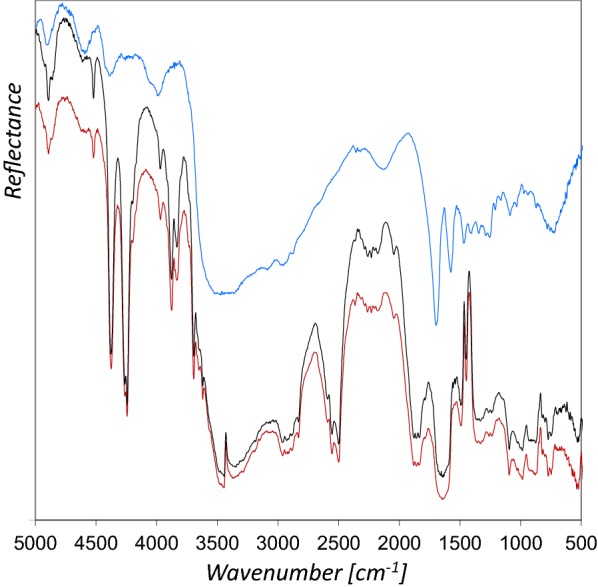
A comparison of the rFTIR spectrum of azurite with glair on parchment (red line) and azurite with glair on aluminum foil (black line) demonstrates that the parchment support of the mockups (blue line) has no influence on the rFTIR spectra

The rFTIR spectra of mockups with one, two and three azurite paint layers do not differ significantly, and a comparison with the mockups on aluminum foil clearly shows that the support does not contribute to the spectra obtained and no spectral features from parchment have to be considered (Fig. 5).

The application of the paints on aluminum foil indicates that Arabic gum paint layers show a greater tendency towards shrinking than glair samples (Fig. 2). This might have implications on the mechanical properties of the illuminations in manuscripts, although Arabic gum was considered as stronger binding medium, and glair as rather brittle [6]. However, the stability of paint layers in illuminations is not only determined by the binding medium, but also by the support.

**Fig. 6:**
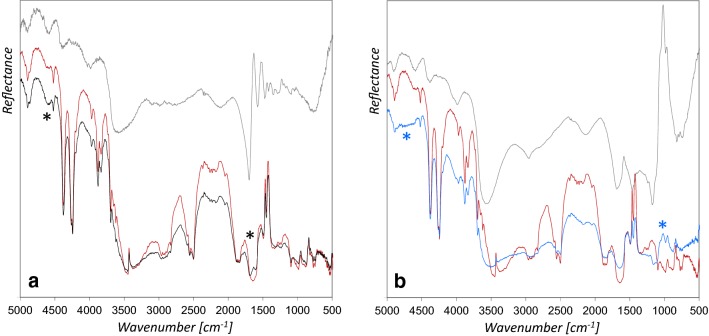
rFTIR spectra of azurite with glair binder (red lines), varnished with **a** glair (black line) and **b** Arabic gum (blue line) with the respective binder references (grey lines). The characteristic features of the varnish materials are indicated with an asterisk

The rFTIR spectra of the areas varnished by using either glair or Arabic gum depict the bands characteristic for the respective materials, regardless which binding medium was used for the pigment. Figure 6 shows the results from azurite with glair as binder and additionally varnished with glair (a), or Arabic gum (b). The results were even more clear, if identical materials were used as binder and varnish. These results demonstrate that rFTIR spectra mainly represent the uppermost layer.

**Fig. 7:**
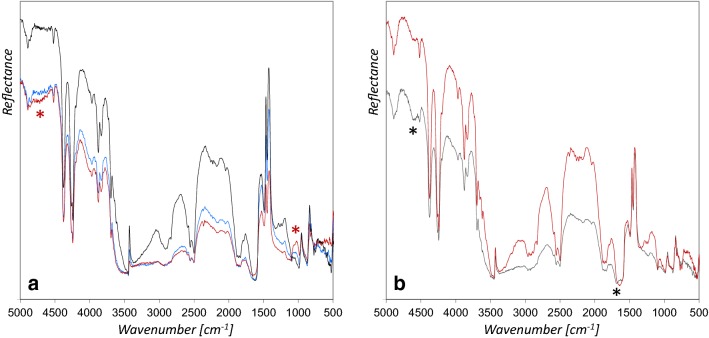
rFTIR spectra of azurite mixed with **a** Arabic gum 20% (black line), 35% (blue line) and 50% (red line) and **b** glair 12% (red line) and 20% (black line). The characteristic features of the binding media are indicated with red asterisks (Arabic gum) and black asterisks (glair)

Using Arabic gum solutions with 35% and 50% dry mass yields gradually enhanced spectral features around 4760 cm^−1^ (ν C–O overtone & combination of ν C–O and δ O–H) and between 1100 and 1000 cm^−1^ (ν C–O) [21], as well as an attenuation of the azurite bands (Fig. 7a). Thompson [6] reports that strong gum solutions were recommended in medieval times to get the maximum depth of blue colors, and in fact the mockups with increased Arabic gum content appeared darker. Moreover, an increase of gloss was observed when a solution of 50% Arabic gum was applied. From a technical point of view, it is not necessary to use solutions with gum contents higher than 20%, and we suppose that illuminators in medieval times only applied higher contents for poor qualities of azurite, or if a glossy surface was explicitly desired.

In the case of glair with 20% dry mass content, slightly enhanced features deriving from the binder were observed around 4600 cm^−1^, corresponding to overtone ν C=O amide I and amide III deformation (ν C–N/δ N–H in plane) [23], and the amide I band around 1690 cm^−1^ [25] (Fig. 7b). However, according to the literature and our preliminary application tests we suppose that illuminators rather used glair undiluted or mixed with water. A comparable content of binder is mainly not expected in historic manuscripts.

The analytical results from the mockups demonstrate the limited possibilities for a characterization of binding media applied for azurite in illuminated manuscripts. In the case of glair the protein contents of pigment/binder mixtures are usually too low for a reliable detection and also a clear distinction from Arabic gum is not possible. Furthermore, it has to be considered that in historic manuscripts thinner paint layers may have been applied and an interference of the protein bands with those of parchment cannot be excluded, as described in the next chapter. On the other hand, Arabic gum could only be detected if applied in very high concentrations or if used as varnish. However, a differentiation of these possible applications by use of rFTIR is hardly possible and would require analyses of cross-sections.

### Manuscripts

**Fig. 8:**
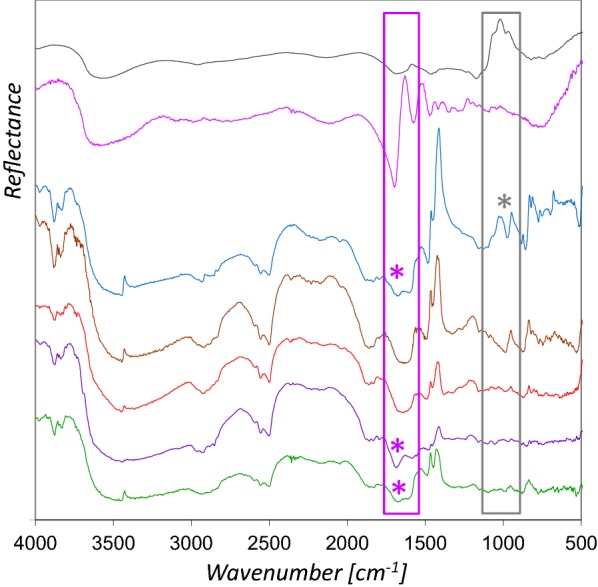
rFTIR reference spectra of Arabic gum (grey) and glair (pink) compared to spectra measured in manuscripts: Cod. slav. 8 (blue), Latin Moamin (brown), Albertinischer Plan (orange), Cod. slav. 3 (purple), and Liederhandschrift B (green). The characteristic features of glair are indicated with a purple asterisk, Arabic gum with a grey

The rFTIR spectra from blue areas in manuscripts investigated are shown in Fig. 8. All exhibit the characteristic bands of azurite, although some are attenuated compared to the spectra from the mockups. Evaluable bands of proteins are recognizable at about 1680 cm^−1^ (Codex slavicus 3 and 8) and 1675 cm^−1^ (Liederhandschrift B), which correspond to the amide I vibration [25], whereas this band is not observable in the spectra obtained from the Latin Moamin and the map (Albertinischer Plan). In both, the reference spectra of parchment and glair used for the mockups amide I causes minima at approximately 1695 cm^−1^, which shows that this band cannot be utilized to distinguish between both types of proteins. Furthermore, this minimum might be shifted to lower wavenumbers in old parchments, e.g. to 1682 cm^−1^ in case of Codex slavicus 8. For these reasons, it is impossible to predict whether the signals derive from the binder or the support. However, the visual inspection of the measurement points with amide I detection rather suggested relatively thin paint layers. In case of the map (Albertinischer Plan), where no protein signal was obtained, the lack of fingerprint bands of the paper support (ν C–O, ν C–C, 1200–1000 cm^−1^) [26] demonstrates that the support does not contribute significantly to the spectrum. As the spectrum shows no indications for any kind of binder it must be presumed that the content is too low for detection by using rFTIR. The fact that the azurite paint layers on the whole object are well preserved even after almost 600 years proves very clearly that the relatively low amount of binder was sufficient.

**Fig. 9:**
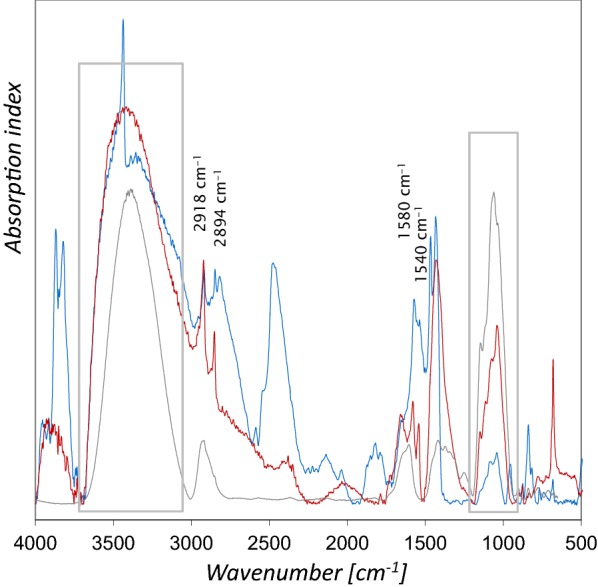
Absorption index spectra from the blue area (blue line) and a light pink area (red line) in Cod. slav. 8 compared to the transmission FTIR reference IRUG CB0084 (grey). The peaks at 2918, 2896, 1580, and 1540 cm^−1^ can be assigned to calcium soap

The spectrum from the blue area in Codex slavicus 8, which corresponds to measurement point 3 in Fig. 1, shows in addition to the bands of azurite and amide I inverted bands in the range of 1200–1000 cm^−1^ (ν C–O) [21] characteristic for gums, which matches with the Arabic gum on parchment reference (Fig. 8), as well as the mockups varnished with Arabic gum. With regard to the results from the mockups it can be presumed, that a gum varnish covers the blue area analyzed in Cod. slav. 8. Moreover, Kramers–Kronig transform of the reflection spectrum yields an absorption index spectrum similar to the IRUG transmission reference spectrum of Arabic gum CB0084 in the range mentioned above, as depicted in Fig. 9. Furthermore, the figure depicts that similar spectral features were detected with even higher intensity in a measurement point of light pink color nearby (measurement point 4 in Fig. 1). A slight surface gloss and some craquelure can be observed, which strongly supports the presumption that a gum varnish was applied. Both spectra exhibit also C–H bands at 2918 and 2894 cm^−1^, as well as COO^−^ antisymmetric stretching bands at 1580 and 1540 cm^−1^, which can be assigned to calcium soaps [27]. One possibility for the presence of these materials could be that soap was added in order to improve the applicability of the paint, which reacted with a calcium compound. However, the C–H vibrations interfere with the ones from proteins and gums, which could potentially be used to differentiate between the binders.

## Conclusions

Azurite was detected in four outstanding illuminated manuscripts on parchment and a colored map on paper, which were investigated non-invasively by rFTIR within the framework of CIMA. As it was impossible to determine whether a proteinaceous or a polysaccharide binder was used, we examined mockups with azurite in combination with either glair or Arabic gum aiming to assess the possibilities for a reliable characterization. Both materials were additionally used to varnish the mockup samples, according to historical sources. The results obtained from the mockups allowed us to state that the used method usually does not enable a reliable characterization of the used binding media, but is capable of determining the varnish materials in certain cases. The investigations further revealed that paint layers in manuscripts are usually more complex, as they may contain additives or reaction products, such as calcium soaps, which may interfere in the spectral ranges, which are important for the characterization of the binding media.

## Abbreviations

CIMA: Centre of Image and Material Analysis in Cultural Heritage
HRSM: Hochschul-Raum Struktur-Mittel
INCDTP: National Research & Development Institute for Textiles and Leather, Bucharest
IRUG: Infrared and Raman Users Group
rFTIR: Fourier Transform Infrared spectrometry in the reflection mode

## References

[1] Eastaugh N, Walsh V, Chaplin T, Siddall R (2008). Pigment compendium, a dictionary and optical microscopy of historical pigments.

[2] Delamare F (2013). Blue pigments: 5000 years of art and industry.

[3] Cennini C. Il libro dell’arte. A cura di F. Frezzato, Neri Pozza, Collana I colibrì; 2009.

[4] Gettens RJ, West Fitzhugh E. Azurite and Blue Verditer. In: Roy A, ed. Artists´ pigments, a handbook of their history and characteristics. Vol 2. London: London Archetype Publications; 1993. p. 23.

[5] Thompson DV (1956). The materials and techniques of medieval paintings.

[6] Thompson DV (1956). The materials and techniques of medieval paintings.

[7] Kroustallis S. Binding media in medieval manuscript illumination: a source research. In: Medieval Colours: between beauty and meaning, Revista de història de arte, no. 1, Serie W. 2011. p. 112-125.

[8] Pessanha S, Manso M, Carvalho ML (2012). Application of spectroscopic techniques to the study of illuminated manuscripts: a survey. Spectrochim Acta B..

[9] Frühmann B, Cappa F, Vetter W, Schreiner M, Father P (2018). Multianalytical approach for the analysis of the Codices Millenarius Maior and Millenarius Minor in Kremsmuenster Abbey, Upper Austria. Herit Sci..

[10] Zaffino C, Guglielmi V, Faraone S, Vinaccia A, Bruni S (2015). Exploiting external reflection FTIR spectroscopy for the in situ identification of pigments and binders in illuminated manuscripts. Brochantite and posnjakite as a case study. Spectrochim Acta A..

[11] Doherty B, Daveri A, Clementi C, Romani A, Bioletti S, Brunetti B, Sgamelotti A, Miliani C (2013). The Book of Kells: a non-invasive MOLAB investigation by complementary spectroscopic techniques. Spectrochim Acta A..

[12] Buti D, Rosi F, Brunetti BG, Miliani C (2013). In-situ identification of copper-based green pigments on paintings and manuscripts by reflection FTIR. Anal Bioanal Chem.

[13] Miliani C, Rosi F, Daveri A, Brunetti BG (2012). Reflection infrared spectroscopy for the non-invasive in situ study of artists’ pigments. Appl Phys A.

[14] Bruni S, Caglio S, Guglielmi V, Poldi G (2008). The joined use of n.i. spectroscopic analyses – FTIR, Raman, visible reflectance spectrometry and EDXRF – to study drawings and illuminated manuscripts. Appl Phys A..

[15] Bicchieri M, Montia M, Piantanida G, Pinzari F, Sodo A (2011). Non-destructive spectroscopic characterization of parchment documents. Vib Spectrosc.

[16] Bruni S, Cariati F, Casadio F, Toniolo L (1999). Identification of pigments on a XV century illuminated parchment by Raman and FTIR microspectroscopies. Spectrochim Acta A..

[17] CIMA is an interuniversity research institution with an interdisciplinary approach aiming at the investigation of cultural heritage and was founded in 2014. http://hrsm.caa.tuwien.ac.at/. Accessed 27 Nov 2018.

[18] Goldsmith JA, Ross SD (1968). The infra-red spectra of azurite and malachite. Spectrochim Acta A..

[19] Price BA, Pretzel B, Lomax SQ, eds. Infrared and Raman Users Group Spectral Database. 2007 ed. Vol. 1 & 2.

[20] Vaculikova L, Plevova E, Vallova S, Koutnik I (2011). Characterization and differentiation of kaolinites from selected Czech deposits using infrared spectroscopy and differential thermal analysis. Acta Geodyn Geomater.

[21] Invernizzi C, Rovetta T, Licchelli M, Malagodi M (2018). Mid and near-infrared reflection spectral database of natural organic materials in the cultural heritage field. Int J Anal Chem..

[22] Aru M, Burgio L, Rumsey MS (2014). Mineral impurities in azurite pigments: artistic or natural selection?. J Raman Spectrosc..

[23] Carlesi S, Becucci M, Ricci M (2017). Vibrational spectroscopies and chemometry for nondestructive identification and differentiation of painting binders. J Chem..

[24] Miguel C, Lopes JA, Clarke M, Melo MJ (2012). Combining infrared spectroscopy with chemometric analysis for the characterization of proteinaceous binders in medieval paints. Chemometr Intell Lab..

[25] Noradi L, Ricciardi P (2019). Non-invasive identification of paint binders in illuminated manuscripts by ER-FTIR spectroscopy: a systematic study of the influence of different pigments on the binders’ characteristic spectral features. Herit Sci..

[26] Polovka M, Polovkova J, Vizarova K, Kirschnerova S, Bielikova L, Vrska M (2006). The application of FTIR spectroscopy on characterization of paper samples, modified by Bookkeeper process. Vib Spectrosc.

[27] Otero V, Sanches D, Montagner C, Vilarigues M, Carlyle L, Lopes JA, Melo MJ (2014). Characterisation of metal carboxylates by Raman and infrared spectroscopy in works of art. J Raman Spectrosc.

